# The legislative backgrounds of workplace health promotion in three European countries: a comparative analysis

**DOI:** 10.1186/s12995-015-0060-y

**Published:** 2015-05-10

**Authors:** Rasa Šidagytė, Maija Eglīte, Anne Salmi, Dovilė Šorytė, Ivars Vanadziņš, Leila Hopsu, Jaana Lerssi-Uskelin, Laima Bulotaitė, Lāsma Kozlova, Svetlana Lakiša, Sigita Vičaitė

**Affiliations:** Occupational Health Centre, Institute of Hygiene, Didžioji str. 22, Vilnius, Lithuana; Department of Occupational and Environmental Medicine, Riga Stradins University, Dzirciema str. 16, Riga, Latvia; Finnish Institute of Occupational Health, Aapistie 1, Oulu, Finland; Institute of Occupational Safety and Environmental Health of Riga Stradins University, Dzirciema str. 16, Riga, Latvia; Finnish Institute of Occupational Health, Topeliuksenkatu 41 a A, Helsinki, Finland; Department of General Psychology, Faculty of Philosophy, Vilnius University, Universiteto str. 9/1, Vilnius, Lithuania; Positive Health Team, Šviesos str. 4B-7, Vilnius, Lithuania

**Keywords:** Workplace health promotion, Occupational health, Enterprise, Legislation

## Abstract

**Background:**

This article investigates the legal database and theoretical basis of workplace health promotion (WHP) in three European countries: Finland, Latvia and Lithuania, and aims to find insights into effective WHP implementation.

**Methods:**

In November 2013, a stakeholders’ survey was carried out. The questionnaire included questions about legal documents and non-legislative measures relevant to WHP, institutions and other bodies/organizations working in the field, WHP conception/definition, and implementation of WHP activities according to the enterprises’ size.

**Results:**

Only Finland has adopted a specific law on occupational health care (separate from occupational safety). ILO conventions No. 161 (Occupational Health Services Convention) and No. 187 (Promotional Framework for Occupational Safety and Health Convention) are ratified only in Finland. In Finland, the Ministry of Social Affairs and Health acts as one ministry, while two Baltic countries have two separate ministries (one for health and another for social affairs). None of the countries has legally approved a definition of WHP. Latvia and Lithuania tend to separate WHP from other activities, whereas Finland integrates WHP into other occupational health and safety elements.

**Conclusions:**

Finland has a more extensive legislative and organizational background to WHP than Latvia and Lithuania. In defining WHP, all the countries refer to the Luxembourg Declaration on Workplace Health Promotion in the European Union. Finland’s practice of integrating WHP into other occupational health and safety elements is important.

## Background

According to the European working conditions survey (EWCS) [1], employees who think that their health or safety is at risk because of their work comprise a quarter in Finland (close to the European average), nearly one third in Lithuania and almost a half in Latvia. Similar figures illustrate the proportion of working people who think that their work mainly affects their health negatively. However, work can also affect the health of working people in a positive way. EWCS revealed that 28.7 percent of Finnish employees think that their work mainly affects their health positively. The Latvian rate for this question was 8.8 percent, and the Lithuanian rate was 6 percent, and the rates of these two countries are around the European average (7.3 percent). Thus employees’ opinions on working conditions and their effect on health seem to be better in Finland than in the two Baltic countries.

Together with other occupational health services, workplace health promotion (WHP) can contribute significantly to the health of the working population. The *Luxembourg Declaration on Workplace Health Promotion in the European Union* [2] defines WHP as “the combined efforts of employers, employees and society to improve the health and well-being of people at work”.

Experts notice that occupational health services are not delivered to all the workers due to gaps in implementation, coverage, content and capacity. These gaps are seen particularly in small enterprises [3]. Countries may have different experiences in developing occupational health services and also different backgrounds for WHP. This article thereafter investigates the legal database and theoretical basis of WHP in three European countries: Finland, Latvia and Lithuania, and aims to find insights into effective WHP implementation.

## Methods

In November 2013, a stakeholders’ survey was carried out using a questionnaire which was prepared and agreed on in all the participating countries. The questionnaire consisted of five sections (each section having several questions): Section A – legal documents and other measures relevant to WHP; Section B – WHP conception/definition; Section C – institutions and other bodies/organizations working in the field of WHP, specific networks and training courses for specialists; Section D – implementation of WHP activities; Section E – research area relative to WHP. The stakeholder institutions were: the Finnish Institute of Occupational Health (Finland), the Institute of Occupational Safety and Environmental Health of Riga Stradins University (Latvia) and the Institute of Hygiene, Occupational Health Centre (Lithuania). Stakeholders chose the appropriate excerpts from the legislative documents for the questionnaire. Then, descriptive analysis of the gathered questionnaires’ content was performed and comparisons made where possible.

The questionnaire elicited the stakeholders’ opinion on the implementation of various WHP activities in the different sized enterprises. In accordance with European Commission Recommendation 2003/361/EC [4] and the Eurostat Glossary [5], a small enterprise is defined as an enterprise that employs fewer than 50 people, a medium-sized enterprise is defined as an enterprise that employs fewer than 250 people and a large enterprise is defined as an enterprise that employs 250 people or more.

## Results

### Legislation concerning WHP

*Council Directive 89*/*391*/*EEC of 12 June 1989 on the introduction of measures to encourage improvements in the safety and health of workers at work* [6] is adapted by all the three countries. The main International Labour Organization (ILO) conventions regarding occupational health issues, *Occupational Health Services Convention No. 161* [7] and *Promotional Framework for Occupational Safety and Health Convention No. 187* [8], are ratified only in Finland. As is seen in Table 1, all the three countries have specific legislations on occupational safety and health issues, including WHP.

**Table 1:** **Most important excerpts of national legislation and other documents connected to WHP**

**Type of legislation**	**Finland**	**Latvia**	**Lithuania**
Laws	1. Occupational Safety and Health Act No. 738/2002 (1958/2002)	Labour Protection Law (2002)	Law on Safety and Health at Work No. IX-1672 (2003/2007)
	2. Occupational Health Care Act No. 1383/2001 (2001)		
Other legislation	Government Decree on the Principles of Good Occupational Health Care Practice, the Content of Occupational Health Care and the Qualifications of Professionals and Experts (2001)	1. Profession Standard for Occupational Safety Specialists (2002)	1. Regulation on Enterprise Occupational Safety and Health Services (2002)
		2. Profession Standard for Occupational Safety Senior Specialist (2002)	2. Regulation on Professional Requirements for Occupational Health Specialists (2008)
			3. Regulation on Professional Development/Training Programme Content Requirements for Occupational Health Specialists (2010)

According to the Finnish *Occupational Safety and Health Act* [9], employers are required to take care of the safety and health of their employees while at work, and to have a policy for actions needed in order to promote safety and health and to maintain employees’ work ability capacity.

The organization of occupational health services in Finland is based on the *Occupational Health Care Act* [10]. This is in line with ILO Convention No. 161. The content of occupational health care includes: making suggestions for action to improve the healthiness and safety of work, to adapt work to the needs of the employee, to maintain and promote employees’ work ability and functional capacity; provision of information, advice and guidance in matters concerning the healthiness and safety of work and the health of the employees.

By enhancing co-operation between employer, employee and occupational health care provider, the Act aims to promote: 1) the prevention of work-related illnesses and accidents, 2) the healthiness and safety of work and the work environment, 3) the health, work ability capacity and functional capacity of employees at different stages of their working careers, 4) the functioning of the workplace community. According to the Act, employers must have a written occupational health care action plan.

The Latvian *Labour Protection Law* [11] includes both the occupational safety and occupational health aspects as the term “labour protection” is defined as “safety and health of employees at work”. The Law defines “labour protection measures” as “legal, economic, social, technical and organisational preventive measures the objective of which is to establish a safe and harmless to health working environment, as well as prevent accidents at work and occupational diseases”. Generally the Law emphasizes that employers are obligated to consult, inform and enforce the participation of employees in the process of occupational safety and health measures.

The Lithuanian *Law on Safety and Health at Work* [12] stresses that employers must ensure employees’ safety and health at work in all work-related aspects. According to this Law, “occupational health” means the prevention of hazardous factors in the work environment, adapting the work environment to the physiological and psychological capabilities of workers, carrying out healthcare of workers, and implementing health strengthening measures.

In all participating countries, the law imposes several occupational safety and health obligations on both employers and employees, as parties in the legal relationship. First of all, the employer is responsible for the employees’ health in general. Directly the specific functions (but not necessarily WHP as itself) are delegated to specialists, who may have a different education and professional titles. In Finland occupational health care providers should put forward initiatives and suggestions to the employer or his representative, on, for example, measures for monitoring the health of employees and different employee groups and for maintaining and promoting their health and work ability [13]. In Latvia, occupational health is included in the obligations of labour protection specialists [14,15] as their training includes various health-related aspects of occupational safety and health. However, health promotion, and specifically WHP, is not directly mentioned in their curricula. In Lithuania, occupational health specialists [16] should perform these functions in relation to WHP [17]: organize healthy lifestyle training, develop health promotion programmes for employees, and arrange their implementation.

Table 2 shows national policy programmes and strategies that come close to WHP in each country.

**Table 2:** **Most important excerpts of national policy programmes and strategies connected to WHP**

**Type of document**	**Finland**	**Latvia**	**Lithuania**
Programmes/strategies specific to workers’ health and safety	Government Resolution. Occupational Health 2015. Development Strategy for Occupational Health Care (2004)	National Strategy on Occupational Safety and Health 2008-2013 (2008) and its action plans for 2008-2010 and 2011-2013	Employee Safety and Health Strategy for 2009–2012 (2009) and its action plans for 2009-2010 and 2011-2012
Programmes/strategies from other areas, or general	1. Programme of Prime Minister Jyrki Katainen’s Government (2011)	1. Public Health Strategy for 2011 – 2017 (2011)	Mental Health Strategy (2007) and its action plan for 2011-2013
		2. Plan of National Development for 2014 – 2020 (2014)	
	2. Finnish Nutrition Recommendations (2014)		
	3. Government Resolution on Development Guidelines for Health-Enhancing Physical Activity and Nutrition (2008)		
	4. On the Move. National Strategy for Physical Activity Promoting Health and Wellbeing 2020 (2013)		
	5. Health Promotion, Government Policy Programme 2007-2011 (2007)		
	6. National Action Plan to Reduce Health Inequalities 2008-2011		
	7. Developing Mass Catering Services. Guidelines by the Working Group to Monitor and Develop Mass Catering Services (2010)		

### Non-legislative measures regarding WHP

Without the legislation (some of it described above), countries apply various non-legislative measures (recommendations, guidelines, other material) prepared by national, local authorities or other bodies. In Finland, the Occupational Safety Card [18] (2003), the Work Capacity Certificate [19] (2006; for students in vocational education) and the Well-being at Work Card [20] (2010; dedicated to everyone) are in use. The *Well*-*being at Work Card* is a one-day training certificate. The topics and contents of the training for the Well-being at Work Card are: some viewpoints on well-being at work; management and well-being at work; the functioning of the work community; health and work ability. In addition to these, there are a great deal of WHP recommendations in Finland.

Latvia has prepared *Health promotion guidelines for local governments* (2011) [21] – some of the recommendations of which are meant for WHP. In Latvia a *Plan for preventive measures* is approved every year. This includes training and information seminars and information materials for occupational safety specialists, employers, employees and others.

Lithuania has several recommendations on different WHP topics for specialists performing occupational health care in enterprises, employers and employees.

### Institutions and other organizations working in the field of WHP

In Finland, the *Ministry of Social Affairs and Health* and the *Ministry of Employment and the Economy* are responsible for policy-making in the area of occupational health and safety (Table 3). The co-ordination and provision of methodological support linked to WHP is responsibility of *Occupational safety and health administrations*. The Administrations enact laws (e.g. Occupational Safety and Health Act, Occupational Health Care Act) and provide supervision and inspection control. The *Finnish Institute of Occupational Health* (*FIOH*) is a research and specialist organization working in the field of occupational health and safety. It organizes qualification training for occupational health and safety professionals and experts. FIOH also organizes and participates in national and international projects and research together with various non-governmental institutions and international universities.

**Table 3:** **Governmental institutions** (**and their activity areas**) **that co**-**ordinate and methodically support WHP activities**

**Type of institutions** **(main functions)**	**Finland**	**Latvia**	**Lithuania**
Ministries	− Ministry of Social Affairs and Health (regulations, enforcement, directions, inspection, monitoring, information, guidelines)	− Ministry of Health (policy-making, recommendations)	− Ministry of Health (policy-making)
		− Ministry of Welfare (policy-making, recommendations)	− Ministry of Social Security and Labour (policy-making)
	− Ministry of Employment and the Economy (legislation on work contracts, annual and other leaves, equity, unemployment)	− Ministry of Education and Science (policy-making, recommendations, professional standards)	
Other institutions at national level	− Occupational Safety and Health Administration (inspection, guidelines and advice relating to health services)	− Institute of Occupational Safety and Environmental Health (recommendations, specialist training)	− Occupational Health Centre, Institute of Hygiene (recommendations, research, specialist training, interventions)
	− Finnish Institute of Occupational Health (FIOH) (training, information, statements, advice, monitoring, recommendations, research, interventions, surveillance, services)		
		− State Labor Inspection (recommendations, inspection, supervision)	
			− State Labour Inspectorate (inspection, supervision, advice)
		− Riga Stradiņš University (specialist training)	
Institutions at regional level	6 Regional State Administrative Agencies (Occupational safety and health administrations) (inspection, guidelines, advise)	Local municipalities (WHP implementation)	Public health offices (WHP implementation)

In Latvia, the *Ministry of Health* and the *Ministry of Welfare* are responsible for the legislation related to occupational health and safety. Both ministries, together with the *Ministry of Education and Science*, the *Institute of Occupational Safety and Environmental Health*, and the *State Labour Inspection* provide various sorts of information and materials that are to some extent attributable to WHP issues. The *Institute of Occupational Safety and Environmental Health* of *Riga Stradiņš University* organize training for specialists and are also known for their participation in national and international research projects and programmes related to WHP. The *State Labour Inspection* executes supervision and inspection control which in some respects is also linked to WHP. The functions of regional institutions belong to *local municipalities* in Latvia. The Latvian *Centre for Disease Prevention and Control* has developed a network of contact persons in local municipalities to co-ordinate health promotion activities locally and their training also includes some aspects of WHP. The Centre itself has developed Health Promotion Guidelines and various information material for local municipalities, which also includes information on health promotion in local companies.

In Lithuania, the responsibility for policy-making concerning occupational health and safety belongs to the *Ministry of Health* and the *Ministry of Social Security and Labour*. The *Occupational Health Centre* (at the *Institute of Hygiene*) and the *Centre for Health Education and Disease Prevention* also provide various recommendations and training for specialists. The *Occupational Health Centre* carries out national and international research and provides expertise, consultation and information in the field of occupational health. *Public health offices* at the municipality level perform trainings and lectures for employees about healthy lifestyles, as well as various other health promotion activities for the employees.

At this moment the two Baltic countries do not have many NGOs that operate in WHP issues or have activities linked to this domain. The Finnish *Centre for Occupational Safety* provides training, information, materials, and development services based on the latest knowledge, and administrates and maitains a register of occupational safety personnel. These activities and services are planned in close co-operation with branch-related employers’ and employees’ organizations in sector groups. In addition, the activities of the Centre are based on the agreements and regulations concluded by Finnish labour market organizations. The Centre provides, for example, one-day training courses, which lead to either the Occupational Safety Card or the Well-being at Work Card.

### Conception and elements of WHP

None of the participating countries has a legally approved definition of WHP. Describing WHP, all partner countries (stakeholder institutions) refer to *The Luxembourg Declaration*, mentioned at the beginning of this article. We can summarize that for implementing WHP at the enterprise level four tool groups are essential: 1) internal political decisions (workplace policies), 2) organizational structures (responsible departments and persons), 3) physical/technical structures (health supporting environment and facilities), and 4) health education (health information and training activities).

In Table 4 we see that in the stakeholders’ opinion, WHP implementation is better in large enterprises, and that medium-sized and especially small enterprises lack WHP activities.

**Table 4:** **Elements of WHP and their implementation according to size of enterprise**

**Elements of WHP**	**Size of enterprise**								
	Finland*			**Latvia**			**Lithuania**		
	**Large**	**Medium**	**Small**	**Large**	**Medium**	**Small**	**Large**	**Medium**	**Small**
1. Specific internal documents approved at the enterprise	P	P	P	P	P	N	P	P	N
2. Initiative of Trade Unions	F	F	P	P	P	N	P	P	N
3. Giving incentives to the employees (contests, premiums, gifts)	P	P	P	F	P	N	P	P	P/N
4. Allocation of funds	F	F	P/N	N	N	N	P	N	N
5. Developing organizational structures in accordance with existing legal acts	F	P	P/N	P	N	N	F	P	N
6. Development of a healthy work environment, e.g. facilities: sports hall, healthy food canteen/shop, relaxation room, park for walking within the grounds of the enterprise, mothers' room etc.	P	P	P	P	N	N	P	P	N
7. Developing healthy psychosocial environment, e.g. conflict management programmes	P	P	N	P	N	N	P	P	N
8. Implementing WHP programmes	P	P	N	P	N	N	P	P	N
9. Dissemination of information about healthy lifestyles, e.g. health education (lectures, seminars), reference material (stands, booklets)	F	P	P	F	P	P	P	P	P
10. Formation of lifestyle skills that sustain and promote health, e.g. physical activity programmes	P	P	P	N	N	N	P	N	N
11. Involving employees and their families in active leisure activities (e.g. sports games for families)	P	P/N	N	P	P	P	P	N	N
12. Developing illness prevention and prophylaxis programmes	P	P/N	N	P	N	N	P	P	N
13. Improving access to health services	P/N	N	N	P	P	N	P	P/N	N

*Finnish information from Aura et al [24].

(F – fully implemented, P – partially implemented, N – not implemented).

Rather then a separate phenomenon in the enterprise, WHP can also be considered as activities integrated into other elements of occupational health and safety. This approach is more characteristic of Finland – WHP is almost “everywhere”. Latvia and Lithuania tend more to separate it from other elements, considering it an independent activity (Table 5).

**Table 5:** **Occupational safety and health activities containing WHP elements**

**Occupational safety and health activities and other enterprise work activities**	**Finland**	**Latvia**	**Lithuania**
1. Occupational risk assessment	+	–	–
2. Preventive health examinations	+	+	–
3. Physical work environment	+	+	+
4. Psychosocial work environment	+	+	+
5. Monitoring of employees’ health and work environment	+	–	–
6. Access to general health services	+	–	–
7. Occupational safety and health policy at the enterprise	+	–	–
8. Rehabilitation (medical, occupational, social)	+	–	–
9. Scientific activities	+	+	+
10. Independent WHP programmes (separate from other activities)	+	+	+
11. Employees consulting	+	–	–

## Discussion

Our study revealed that there are differences between the countries as regards the backgrounds of WHP implementation. Finland has a more extensive legislative background for occupational health services and WHP implementation. All three countries have specific legislations and other documents on occupational safety and health issues, including WHP, but Finland is the only country that has adopted a specific law on occupational health care (separate to occupational safety). ILO conventions regarding occupational health issues, *Occupational Health Services Convention No. 161* and *Promotional Framework for Occupational Safety and Health Convention No. 187*, are ratified only in Finland.

Another feature of Finland’s system is that the Ministry of Social Affairs and Health in this country acts as one ministry while the two Baltic countries have two separate ministries (one for health and another for social affairs). All these issues are important for organizing occupational health care. The Finnish model seems to be more extensive. Surveys show that working conditions and their affect on health according to employees’ opinions, is better in Finland than in the two Baltic countries – this could be the result of the extensive legislative system. However, in saying this, we do not claim that legislation alone determines success. But legislation should be adequate to build a substantial background for occupational health services and WHP implementation. The governments of Latvia and Lithuania should consider including the provisions of ILO conventions mentioned above in their national legislation.

It is important to mention that in Lithuania, only bigger enterprises (with more than 100 or 200 employees, depending on the economic activity (i. e. risk level)), have to employ/hire occupational health specialists. In both Latvia and Lithuania in some cases (small companies) the employer himself is allowed to act as the occupational safety and health specialist – he only has to undergo basic level training for this. This is a problem in the light of WHP, because most employees work in smaller companies and this means that providing them with WHP services (and the quality of these services) depends on the employers’ education, interests and resources.

The survey made it obvious that none of the participating countries has a legally approved definition of WHP. However, this is not necessary, as all three countries refer to the Luxembourg Declaration. Actually, all these countries are considered members of the *European Network for Workplace Health Promotion* (*ENWHP*) [22], but the two Baltic countries are currently not active. In our opinion, this situation shows the lack of the Baltic countries’ government’s attention to international collaboration in the occupational health area. Another actual problem that stakeholders mentioned is that the WHP training for specialists (dedicated directly to WHP) is very poor. Even in Finland, WHP is at the moment only one aspect of specialists’ training. The current situation generally calls for a more integrative approach and understanding of WHP and the safety of workers [23].

To effectively contribute to the health of workers, WHP should be organized along with other occupational health and safety activities (occupational risk assessment, preventive health examinations etc.) which are all of equal importance. We noticed that sometimes WHP can even be integrated into these other activities. Latvia and Lithuania tend to treat WHP as a more independent activity, whereas Finland often integrates WHP into other occupational health and safety elements. Remembering the figures on how work affects employees’ health in each country, we can state again that the Finnish approach to WHP may be more effective.

A limitation of our survey is that the results are based only on stakeholders’ opinions. There is a need to carry out an enterprise survey on the existing practical level and the needs for WHP activities. Since employers’ motivation is essential for implementing WHP, it is necessary to find out what motivates them and what obstacles they face in this area. We also lack knowledge regarding the employees’ WHP needs. Thus, recommendations on how to most effectively implement WHP should be prepared for decision-makers, employers, occupational and public health specialists, and human recourse specialists.

## Conclusions

1. All three countries have specific legislation on occupational safety and health issues but Finland has a more extensive legislative and organizational background to WHP.
2. None of the investigated countries has a legally approved definition of WHP, but all refer to the Luxembourg Declaration.
3. WHP realization may have different approaches. Finland’s practice on integrating WHP into other occupational health and safety elements is important.
